# Impaired discrimination of negative facial emotions in pure motor ALS

**DOI:** 10.1007/s00415-026-14241-x

**Published:** 2026-11-10

**Authors:** Antonia Meyer, Anna Semenkova, Steven Wezel, Markus Weber, Kathi Schweikert, Regina Schläger, Janina Wendebourg, Olivier Piguet, Manfred Berres, Nathalie Braun, Dorothée Lulé, Marc Sollberger

**Affiliations:** 1 Memory Clinic, Department of Dementia Diseases, University Hospital of Geriatric Medicine FELIX PLATTER and University of Basel, Basel, Switzerland; 2 Department of Neurology and Department of Clinical Research, University Hospital Basel, Basel, Switzerland; 3 Faculty of Psychology, University of Basel, Basel, Switzerland; 4 HOCH Health Ostschweiz, Kantonsspital St. Gallen Muskelzentrum/ALS Clinic, St. Gallen, Switzerland; 5 School of Psychology and Brain and Mind Centre, The University of Sydney, Sydney, NSW Australia; 6 Department of Mathematics and Technology, University of Applied Sciences Koblenz, Koblenz, Germany; 7 Department of Neurology, Ulm University Medical Center, Ulm, Germany

**Keywords:** Amyotrophic lateral sclerosis, Pure motor ALS, Frontotemporal dementia, Facial emotion recognition, Social cognition

## Abstract

**Background and Objectives:**

Emotion recognition deficits in amyotrophic lateral sclerosis (ALS) remain debated. We investigated whether patients with pure motor ALS (without cognitive and behavioural disturbances) and without clinically relevant mood disturbances are impaired in discriminating negative facial emotions, using a test without ceiling effects.

**Methods:**

This cross-sectional study included 35 patients with pure motor ALS, based on the revised Strong criteria and operationalised using the Edinburgh Cognitive and Behavioural ALS Screen (ECAS), without clinically relevant mood disturbances, alongside 35 age-, sex-, and education-matched cognitively and psychiatrically healthy controls. All participants completed a shortened five-emotion version of the Facial Emotion Intensity Rating Test – Congruent and Incongruent and the Facial Sex Matching Task (FSMT), a brief screening measure of non-emotional facial discrimination.

**Results:**

Pure motor ALS patients showed poorer discrimination among negative facial emotions than healthy controls (*p* = 0.011, Cohen’s d = 0.63). The group difference did not differ significantly across anger, fear, sadness, and disgust (*p* = 0.178). Similarly, the group difference in discrimination among negative emotions did not differ significantly from that in discrimination between negative emotions and happiness (*p* = 0.459). The two groups did not differ significantly on the FSMT.

**Conclusion:**

Discrimination of negative facial emotions was impaired in patients with pure motor ALS, suggesting that classification based on ECAS screening may underestimate social-cognitive involvement. Our findings are compatible with the concept of an ALS–frontotemporal spectrum disorder. Whether impaired emotion discrimination relates to additional cognitive or behavioural impairment remains to be determined. The clinical utility and reliability of our measure require further study.

**Supplementary Information:**

The online version contains supplementary material available at https://doi.org/10.1007/s00415-026-14241-x.

## Introduction

Amyotrophic lateral sclerosis (ALS) is a neurodegenerative disease that affects motor neurons in the brain and the spinal cord. Although ALS was once considered a purely motor neuron disorder, it is now well recognised that patients may exhibit a wide range of concurrent cognitive and/or behavioural symptoms [1]. Up to 50% of patients present with cognitive and/or behavioural deficits, and 10–15% meet the diagnostic criteria for frontotemporal dementia (FTD) [2, 3]. The revised Strong criteria [3] classify cognitive and behavioural involvement in ALS under the term ALS–frontotemporal spectrum disorder (ALS-FTSD), which comprises ALS with cognitive impairment (ALSci), ALS with behavioural impairment (ALSbi), ALS with both cognitive and behavioural impairment (ALScbi), and ALS-FTD; patients without such involvement are referred to here as having pure motor ALS.

Cognitive functions that are typically impaired in ALS include language, executive functions, and social cognition [4]. In contrast to language and executive functions, social cognition is still rarely assessed in clinical routine [5]. Key domains of social cognition include the ability to recognise emotions (emotion recognition, ER) and the capacity to infer the thoughts and feelings of others, referred to as Theory of Mind (ToM) [6]. Assessing social cognition in ALS is crucial, not only because social-cognitive deficits are common across the disease spectrum [4], but also because these deficits can severely impact the quality of life of patients and their carers [7]. Importantly, ER capacity can be modulated by affective states, such as depression [8], and depressive symptoms, affect approximately one third of ALS patients [9]; affective status, therefore, requires careful consideration when examining social-cognitive functions in this population.

Established cognitive screening instruments for ALS cover social cognition only to a limited extent: the Edinburgh Cognitive and Behavioural ALS Screen (ECAS) [10] includes a ToM task within the executive function domain, but no ER task. A meta-analysis has shown that ER and ToM are impaired to a similar extent in patients with ALS, both with medium effect sizes[11]. However, ER and ToM differ fundamentally in their cognitive architecture. ToM is a higher-order cognitive function that is tightly associated with executive functions [12–14], a domain assessed in all established ALS-specific cognitive screening instruments [10, 15]. In a large ALS cohort, ToM partially overlapped with executive measures, whereas facial ER did not [16]. Likewise, across studies in ALS, the recognition of basic emotions was not correlated with executive dysfunction, whereas findings on cognitive ToM may be confounded by it [14]. Consequently, if a component representing social cognition is to be incorporated into ALS cognitive screening, assessing ER offers an advantage over ToM, reducing the probability that a deficit reflects executive dysfunction.

Findings regarding ER impairment across the different clinical presentations of ALS are conflicting [17]. Varying task demands, emotional categories, and ALS phenotypes across different studies may account for these inconsistencies [17]. Overall, ALS patients tend to show impairments in facial ER, particularly for negative basic emotions [17]. These deficits are most pronounced in ALS-FTD [17], where they are at least as severe as in behavioural variant FTD (bvFTD) [18], a syndrome characterised by early and prominent ER deficits [19]. By contrast, the evidence on whether ER deficits are tied to cognitive-behavioural status is inconsistent: some studies report ER deficits confined to patients with cognitive or behavioural impairment (with the overall ALS group unimpaired), whereas others find a similar proportion of ER impairment regardless of cognitive-behavioural status [17].

Comparisons across studies are further complicated by differences in how the absence of cognitive and behavioural involvement was defined: most studies excluded only patients with dementia, in particular FTD [17], and some referred to the remaining non-demented patients as ‘pure ALS’, although such groups may include patients with ALSci, ALSbi, or ALScbi [18]. Studies that classified patients according to the revised Strong criteria [3], thereby excluding cognitive impairment (defined by executive or language dysfunction) and behavioural impairment, consistently found poorer overall ER, but differed in the emotions affected. Palumbo et al. [20] (n = 49) reported poorer overall facial ER relative to healthy controls (HCs), with significant deficits for sadness and fear only. In contrast, Castelnovo et al.[21] (n = 26) found lower overall scores on the Comprehensive Affect Testing System (CATS), with disgust as the only individual emotion affected. However, neither study rules out that the ER deficit reflects unrecognised cognitive impairment or depressed mood. Under the revised Strong criteria, patients with deficits in other cognitive domains may have been classified as unimpaired. Depression, which may modulate ER capacity [8], was excluded only at the level of major depression [20] or was assessed without a stated criterion [21]. In addition, both studies used tasks with full-intensity facial expressions (the Ekman 60 Faces Test, EK-60F [20], and the CATS [21]), which carry a risk of ceiling effects [22]. Whether ER is impaired in patients with ALS in whom cognitive impairment in all domains and clinically relevant depressive symptoms have been excluded, therefore, remains open. Addressing this question requires a study that combines three features: a stricter classification of pure motor ALS requiring normal performance in all ECAS cognitive domains, including those not specific to ALS; the exclusion of clinically relevant depressive symptoms using a predefined cut-off on an ALS-specific instrument; and an ER task sensitive to subtle impairments, that is, free of ceiling effects.

Accordingly, we examined patients with pure motor ALS who performed normally in all cognitive domains of the ECAS [23] and had no clinically relevant depressive symptoms on the ALS-Depression-Inventory (ADI-12) [24, 25], and used a shortened five-emotion version of the Facial Emotion Intensity Rating Test – Congruent and Incongruent (FEIRT-CIC) [26], a facial emotion discrimination test without ceiling effects [22]. To assess potential group differences in non-emotional facial discrimination, we administered the Facial Sex Matching Task (FSMT), a brief task developed to match the FEIRT-CIC in stimulus material, as a screen for differences in basic facial processing. Both tasks use morphed Ekman faces (emotional expressions of varying intensity in the FEIRT-CIC, neutral expressions in the FSMT) from the Facial Expressions of Emotion – Stimuli and Tests (FEEST) battery [27].

We hypothesised that, even in the absence of overt cognitive impairment, patients with pure motor ALS would show impaired ER performance relative to HCs for negative basic emotions. As a secondary aim, we examined whether any impairment extends beyond discrimination within negative emotions to discrimination between valences, that is, of happiness from negative emotions.

## Methods

### Participants

Patients and HCs were examined between November 2018 and December 2024. Thirty-five patients with probable or definite ALS according to the revised El Escorial criteria [28], classified as pure motor according to the revised Strong criteria [3], were recruited from German-speaking neuromuscular centres in Switzerland (Basel and St. Gallen) and Germany (Ulm). Of the 35 patients with ALS, 11% (n = 4) were recruited in Basel, 26% (n = 9) in St. Gallen, and 63% (n = 22) in Ulm (for detailed information about the number of screened and excluded ALS patients see the participant flow diagram in Supplementary Fig. S1; for additional clinical characteristics, see Supplementary Table S1). Sample size was determined by a power analysis based on a one-sided test (see Supplementary Material A). To ensure diagnostic accuracy, a one-year follow-up was conducted, which confirmed the diagnosis of ALS in all patients.

Further inclusion criteria were fluency in written and spoken German or Swiss German, the ability to read and understand text, and the capacity to provide informed consent. Additionally, a reliable informant with at least monthly contact with the patient was required. Exclusion criteria included cognitive and/or behavioural impairments as defined by the German or Swiss German versions of the Edinburgh Cognitive and Behavioural ALS Screen (ECAS) [23], as well as any factors that could influence clinical presentation and ER capacity. These included clinically significant brain pathologies (e.g., severe vascular brain lesions), severe visual impairments, clinically relevant depressive symptoms defined by a score of ≥29 on the ADI-12, an instrument developed for ALS that avoids items confounded by motor symptoms [24, 25], other psychiatric diseases as defined by the International Statistical Classification of Diseases and Related Health Problems (ICD-10) [29], severe somatic diseases, a lifetime history of substance abuse, current invasive ventilation, or general anaesthesia within the last three months.

Thirty-five HCs without cognitive or psychiatric disorders, matched to the patients in terms of age, sex, and education, were recruited from the participant pool of the Memory Clinic Basel, Switzerland. Matching was performed manually at the individual level: each patient was matched 1:1 to one HC from this pool, with exact matching for sex and matching as close as possible for age and years of education. Inclusion criteria were fluency in Swiss German/German, self-reported good health, and written informed consent. Exclusion criteria for HCs included: (i) cognitive impairment, operationalised as (a) self-reported and/or informant-reported cognitive decline, the latter defined by a score of >3.29 on the Informant Questionnaire on Cognitive Decline in the Elderly (IQCODE) [30], and/or (b) a cognitive screening score below the 10th percentile on the Montreal Cognitive Assessment (MoCA) [31, 32]; (ii) signs of depression, defined as a Beck Depression Inventory (BDI) [33] score >9 [34] for subjects under 65 years or a Geriatric Depression Scale (GDS) score >5 for subjects 65 years and older [35]; and (iii) any other factors with a potentially negative influence on ER (such as general anaesthesia within the last three months, specific medications, or neurological and psychiatric diseases).

Recruitment began in Ulm in November 2018 with approval of the local ethics committee (No. 19/12), and in Basel and St. Gallen in 2019 after approval by the Ethics Committee of Northwestern and Central Switzerland (EKNZ 2019–00213); all study procedures complied with the Declaration of Helsinki.

### Tests and procedure

All participants completed the following tasks in a single session, administered in the order described below.

Facial Sex Matching Task* (*FSMT): To assess participants’ ability to discriminate facial features independent of emotional content, we administered the computer-based FSMT [36] using morphed Ekman faces with neutral expressions from the FEEST [27]. Participants were presented with pairs of faces (either of the same sex or of different sexes) side by side and were asked to indicate whether the two faces were of the same sex or not. After one practice trial, six test trials followed. Each stimulus remained on the screen until a response was given, and the task was untimed. The FSMT score represents the total number of correct responses (0–6), where higher scores reflect better performance. Psychometric data indicate modest reliability (test–retest r = 0.68; Cronbach’s α = 0.55–0.62; intraclass correlation coefficient = 0.46) and limited evidence for validity[36]. See Supplementary Materials B and E for further details.

Facial Emotion Intensity Rating Test–Congruent and Incongruent (FEIRT-CIC): After the FSMT, participants completed a shortened five-emotion version of the computer-based FEIRT-CIC (FEIRT-CIC-SF), as proposed in the original study to reduce administration time [26]. Five basic emotions (anger, disgust, fear, happiness and sadness) were presented for both sexes at threshold intensity levels, as previously established [22]. Each face was shown five times in succession, each time with a different emotion label displayed above it: once for rating the congruent emotion (e.g., rating sadness when a sad face is shown) and four times for rating the incongruent emotions. The emotion labels were presented in the same fixed order for all faces (anger, sadness, fear, happiness, disgust), so that the position of the congruent rating varied with the facial emotion. The 10 faces (5 emotions × 2 sexes) were presented in a fixed, pseudo-randomised order in which faces showing the same emotion never followed each other directly, resulting in 50 trials. Participants rated how intensely the face expressed the emotion named by the label by selecting one of seven unnumbered circles increasing in size from left to right, with the endpoints labelled “no [emotion]” and “maximum [emotion]” (e.g., “no anger”, “maximum anger”); responses were scored from 1 to 7 (for an illustration, see Supplementary eFigure 1 in Chiu et al. [26]).

Test administration was standardised across sites: all tests were administered in a quiet room using the same German-language version of the computerised task (E-Prime 1.2; Psychology Software Tools, Pittsburgh, PA) on the same laptop model (15-inch), with scripted instructions. Examiners at all sites were instructed to introduce the content of the test before the computerised task started. Practice trials preceded testing; the task was untimed and no feedback was given [22, 26]. If motor impairment prevented a patient from operating the mouse, the examiner entered the response indicated by the participant.

Edinburgh Cognitive and Behavioural ALS Screen (ECAS): Patients with ALS completed the German or Swiss German versions of the ECAS [23, 37], interpreted with the age- and education-adjusted cut-offs of Loose et al. [23].

Behaviour was assessed with the ECAS Behaviour Screen – Carer Interview [10], completed by an informant with at least monthly contact with the patient. In line with the revised Strong criteria [3], patients with apathy or with at least two other behavioural symptoms (such as disinhibition, loss of sympathy or empathy, perseverative or compulsive behaviour, or hyperorality) were classified as ALSbi and excluded (for behavioural symptoms in the included patients, see Table 1). The ECAS Behaviour Screen covers the five behavioural features of bvFTD according to Rascovsky et al. [38], as does the informant-rated Behavioural Dysfunction Questionnaire (BDQ) [39], which was used at baseline in one patient without baseline ECAS behavioural data; in this patient, the BDQ indicated no behavioural impairment, and the ECAS Behaviour Screen at follow-up was unremarkable. In the present study, pure motor ALS was defined as ALS without ALSci, ALSbi, ALScbi, or ALS-FTD according to the revised Strong criteria [3], with two additional requirements: normal performance in all ECAS cognitive domains, including the ALS-non-specific domains memory and visuospatial functions, and the absence of clinically relevant depressive symptoms (defined by a score of ≥29 on the ADI-12 [24, 25]) and of other psychiatric disorders.

**Table 1:** Demographic and clinical characteristics of study participants by diagnostic group (N = 70)

Characteristic	Pure motor ALS n = 35	HC n = 35	Test (df)	*p*
*Age (years)*	62.17 ± 8.64	62.69 ± 8.26	−0.25 _(67.86)_^a^	0.800
*Sex (female/male)*	13/22	13/22	matched^b^	—
*Education (years)*	13.61 ± 2.98	14.00 ± 2.84	−0.55 _(67.85)_^a^	0.581
*ECAS total (0–136)*	114.69 ± 7.77	115.71 ± 9.75	−0.49 _(64.77)_^a^	0.627
ECAS Memory (0–24)	17.51 ± 2.73	18.91 ± 3.34	−1.92 _(65.40)_^a^	0.059
ECAS Visuospatial (0–12)	11.97 ± 0.17	11.91 ± 0.28	1.02 _(55.40)_^a^	0.311
ECAS Language (0–28)	26.74 ± 1.52	27.03 ± 1.04	−0.92 _(60.17)_^a^	0.363
ECAS Fluency (0–24)	19.14 ± 2.96	20.46 ± 3.22	−1.78 _(67.53)_^a^	0.080
ECAS Executive Functions (0–48)	39.31 ± 3.62	37.40 ± 6.71	1.49 _(52.25)_^a^	0.143
*Facial Sex Matching Task (0–6)*	5.20 ± 1.05	5.31 ± 0.93	W = 585^c^	0.725
*Any behavioural symptom, n/N (%)*^*d*^	2/34 (6%)	n/a	—	—
*ALS-Depression-Inventory, ADI-12 (12–48)*	20.06 ± 4.30	n/a	—	—
ADI-12 < 22 (inconspicuous), n/N (%)	18/35 (51%)	n/a	—	—
ADI-12 22–28 (mild to moderate depressive symptoms), n/N (%)	17/35 (49%)	n/a	—	—
*Depression screening — positive screens, n/N (%)*				
ADI-12 ≥ 29 (patients)^e^	0/35 (0%)	n/a	—	—
GDS > 5 (HC)	n/a	0/14 (0%)	—	—
BDI > 9 (HC)	n/a	0/21 (0%)	—	—
*Time from symptom onset to assessment (years)*^*f*^	1.96 ± 1.53	n/a	—	—
*ALS Functional Rating Scale–Revised (0–48)*	36.94 ± 6.78	n/a	—	—

*ALS* amyotrophic lateral sclerosis, *pure motor ALS* ALS without cognitive and behavioural impairment according to the revised Strong criteria, as assessed with the ECAS, with normal performance in all ECAS cognitive domains, and without clinically relevant depressive symptoms or other psychiatric disorders. *HC* healthy controls, *ECAS* Edinburgh Cognitive and Behavioural ALS Screen, *n/a* not applicable. Values are mean ± SD unless otherwise indicated

^a^Welch’s t test (two-sided); ^b^exactly matched by design; ^c^Wilcoxon rank-sum test. ^d^Any behavioural symptom among the 34 patients with baseline ECAS behavioural data (loss of sympathy or empathy, n = 1; hyperorality, n = 1); ^e^ADI-12 = ALS-Depression-Inventory; *GDS* Geriatric Depression Scale, *BDI* Beck Depression Inventory. For depression screening, n/N denotes the number of positive screens (ADI-12 ≥ 29, GDS > 5, BDI > 9) / number assessed using the respective instrument. ADI-12 categories according to Kübler et al.[25]. ^f^n = 33

HCs completed the ECAS to provide a clinical baseline for comparison, while their cognitive health was verified via the MoCA and IQCODE as detailed above.

### Data analysis

#### Comparison of FEIRT-CIC-SF rating scores between groups

In line with the literature on ER deficits in patients with ALS [17, 20] and other neurodegenerative diseases [40, 41], our primary analysis focused on the discrimination among the four negative emotions (anger, fear, disgust and sadness). Post-hoc analyses of the single emotion scores were planned if the primary result was significant.

As a secondary analysis, we examined the ability to distinguish happiness, a positively valenced emotion, from the four negative emotions combined. This analysis was designed to test whether any observed ER impairment reflects a selective deficit in within-category discrimination of negative emotions, or whether it extends to cross-valence discrimination more broadly. Surprise was not part of the shortened task, because its ambiguous valence[42] precludes its assignment to either negative or positive emotions.

Scores were calculated as follows:

- Congruent rating score for negative emotions (NET-Con; NET = negative emotion total): the mean of the congruent ratings for both female and male facial stimuli across the four negative emotions (anger, fear, disgust and sadness) (2 sexes × 4 emotions = 8 ratings).
- Incongruent rating score for negative emotions (NET-Incon): the mean of the incongruent ratings for both female and male facial stimuli across the four negative emotions, with the three ratings on the other negative emotion labels per stimulus (2 sexes × 4 emotions × 3 ratings = 24 ratings).
- Contrast rating score for negative emotions (NET-Contrast): calculated by subtracting the NET-Incon score from the NET-Con score.
- Contrast rating score for happiness (Happiness-Contrast): calculated by subtracting the Happiness-Incon score (the mean of the incongruent ratings on the four negative emotions for facial stimuli portraying happiness) from the Happiness-Con score (the mean of the congruent ratings for facial stimuli portraying happiness).
- Contrast rating scores for each negative emotion, for example Sadness-Contrast: calculated by subtracting the Sadness-Incon score (the mean of the incongruent ratings on the three other negative emotions for facial stimuli portraying sadness) from the Sadness-Con score (the mean of the congruent ratings for facial stimuli portraying sadness).

Prior to analysis, data distributions and potential outliers were visually inspected for all scores. In cases where outliers were identified, their influence on the results was assessed using standard influence diagnostics. The primary analysis compared the NET-Contrast score between patients and HCs using Welch’s t test; although participants were matched individually, groups were analysed as independent samples. All tests were two-sided; group differences in the FEIRT-CIC-SF scores are reported with 95% confidence intervals.

Trial-level ratings of the negative-emotion stimuli were additionally analysed with a linear mixed-effects model including group, congruency, and their interaction as fixed effects, random intercepts for participant and stimulus, and a by-participant random slope for congruency, which improved model fit (likelihood-ratio test, χ^2^(2) = 47.1, p < 0.001; Satterthwaite degrees of freedom); in this model, the group × congruency interaction corresponds to the group difference in the NET-Contrast score. In post-hoc sensitivity analyses of potential site effects, NET-Contrast scores were compared between the three recruitment sites (Welch’s ANOVA); as the patients from Basel differed from those of the other two sites, the primary comparison was repeated for patients from Ulm only (the only site with a sample large enough for a separate comparison, n = 22) and for all patients except those from Basel. Congruent and incongruent ratings were compared separately between groups.

Individual emotion contrast scores were compared post hoc using Welch’s t tests (uncorrected and Holm-adjusted). Whether the group difference varied across the four negative emotions was tested by the group × emotion interaction in a 2 × 4 mixed ANOVA with emotion (anger, fear, sadness, disgust) as within-subject factor and group (ALS vs. HC) as between-subject factor (Greenhouse–Geisser-corrected where appropriate). The Happiness-Contrast score was compared between patients and HCs using Welch’s t test. Whether the group difference differed between negative emotions and happiness was tested by the group × valence interaction in a 2 × 2 mixed ANOVA with valence (NET-Contrast vs. Happiness-Contrast) as within-subject factor and group as between-subject factor. Demographic characteristics, ECAS scores, and FSMT scores were compared between groups using Welch’s t tests or Wilcoxon rank-sum tests, as appropriate. Effect sizes are reported as Cohen’s d with 95% confidence intervals; for the two previous cohorts, d was derived from published summary statistics. The significance level was α = 0.05. Analyses were performed in R 4.3.3 (lme4, lmerTest, afex).

### Use of large language models

A large language model (Claude, Anthropic) was used to assist with language editing, manuscript preparation, and the additional statistical analyses of the revision. All analyses were verified independently by the authors, who take full responsibility for the scientific content, the interpretations, and the final manuscript.

## Results

**Fig. 1:**
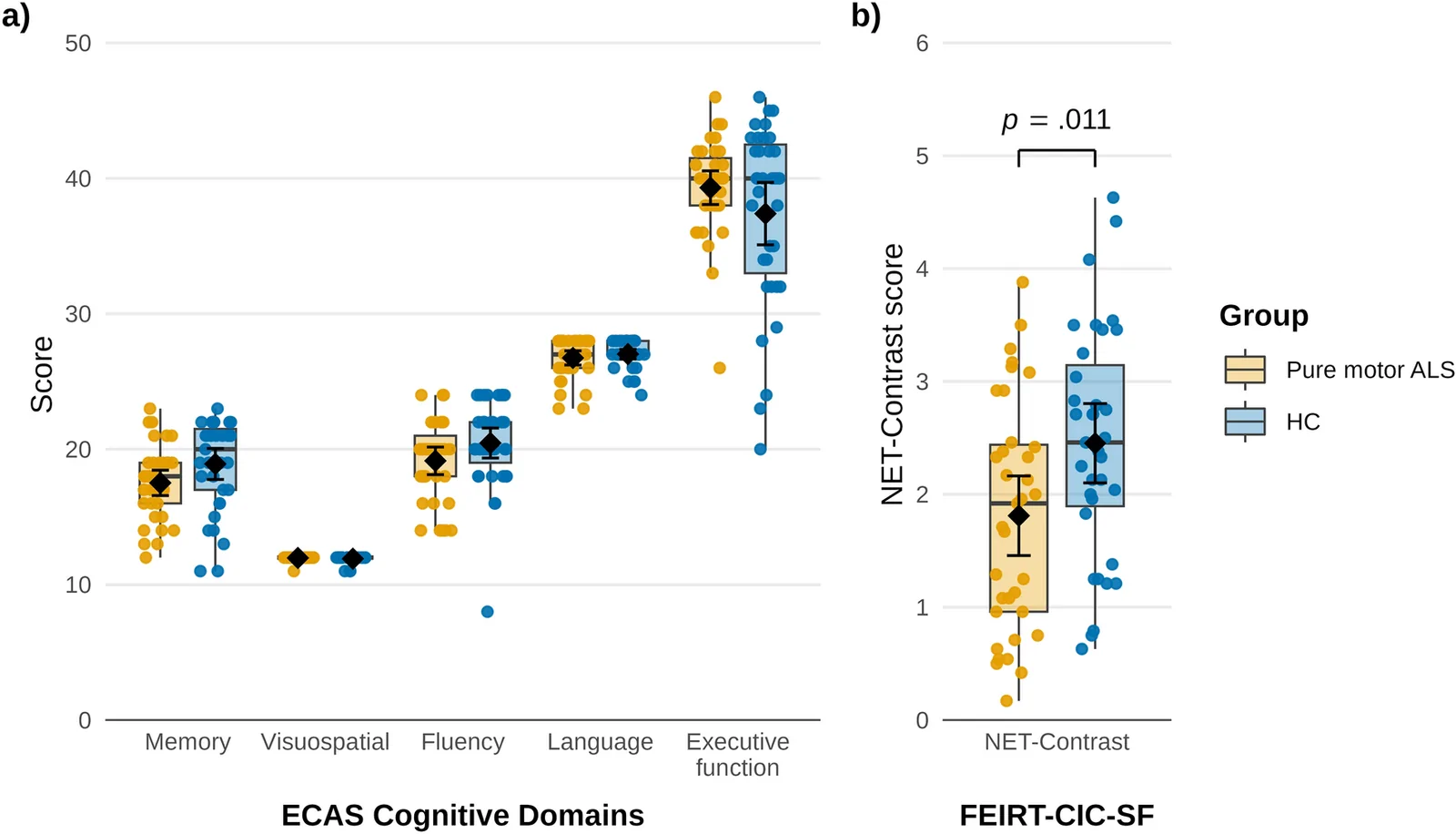
Comparison of **a** the five cognitive domain scores of the Edinburgh Cognitive and Behavioural ALS Screen (ECAS) and **b** the contrast rating score for negative emotions (NET-Contrast) of the shortened five-emotion version of the Facial Emotion Intensity Rating Test – Congruent and Incongruent (FEIRT-CIC-SF) between pure motor amyotrophic lateral sclerosis (ALS) patients and healthy controls (HC). Dots represent individual participants (horizontal jitter). Boxes indicate the median and interquartile range, and whiskers extend to the most extreme values within 1.5 × the interquartile range; black diamonds and error bars represent group means and two-sided 95% confidence intervals. The p-value in **b**) refers to the two-sided Welch’s t-test

The groups, matched for age, sex, and years of education, did not differ significantly in the ECAS total or domain scores (Table 1; Fig. 1a). No participant met the exclusion criterion for clinically relevant depressive symptoms on their respective screening instrument. Of the 35 patients, 10 (29%) had bulbar involvement at onset, 7 (20%) used non-invasive ventilation, and 27 of the 31 with available medication records (87%) were taking riluzole; a C9orf72 repeat expansion was documented in 2 patients, and genetic status was not assessed in the remaining 33 (Supplementary Table S1).

There were no missing FEIRT-CIC-SF, FSMT, or ECAS cognitive data, and the complete sample was included in all analyses. Visual inspection and influence diagnostics revealed no influential outliers in any of the FEIRT-CIC-SF scores.

### Ability to distinguish between negative emotions

NET-Contrast scores were significantly lower in patients than in HCs (Welch’s t test, t(68.0) = −2.62, p = 0.011, mean difference = −0.64, 95% CI [−1.13, −0.15]; Table 2), corresponding to a medium effect size (Cohen’s d = 0.63; Fig. 1b). As expected for this balanced design, the trial-level linear mixed-effects model reproduced this result (group × congruency interaction: β = −0.64, SE = 0.24, p = 0.011, 95% CI [−1.12, −0.16]). In post-hoc sensitivity analyses, NET-Contrast scores of patients differed between recruitment sites, with the highest scores in the four patients from Basel (Supplementary Material D); the group difference was preserved when only patients from Ulm (n = 22; p = 0.014, Cohen’s d = 0.69) or all patients except those from Basel (n = 31; p = 0.001, Cohen’s d = 0.83) were compared with the HCs.

**Table 2:** Performance on the FEIRT-CIC-SF test by diagnostic group (*N* = 70)

Score	Pure motor ALS n = 35	HC n = 35	Mean difference (95% CI)	d (95% CI)	p
*NET-Contrast*	1.81 ± 1.03	2.45 ± 1.02	−0.64 [−1.13, −0.15]	0.63 [0.14, 1.11]	0.011
*NET-Con (congruent ratings)*	4.38 ± 0.95	4.72 ± 0.92	−0.34 [−0.78, 0.11]	0.36 [− 0.11, 0.83]	0.137
*NET-Incon (incongruent ratings)*	2.57 ± 0.96	2.27 ± 0.68	0.31 [−0.09, 0.71]	−0.37 [− 0.84, 0.11]	0.128
*Anger-Contrast*	2.70 ± 1.55	3.61 ± 1.14	−0.91 [−1.56, −0.27]	0.67 [0.19, 1.15]	0.006
*Disgust-Contrast*	−0.05 ± 1.50	0.25 ± 1.66	−0.30 [−1.05, 0.46]	0.19 [− 0.28, 0.66]	0.438
*Fear-Contrast*	2.57 ± 1.83	3.60 ± 1.57	−1.04 [−1.85, −0.23]	0.61 [0.13, 1.09]	0.013
*Sadness-Contrast*	2.02 ± 1.07	2.35 ± 1.52	−0.32 [−0.95, 0.31]	0.25 [− 0.23, 0.72]	0.308
*Happiness-Contrast*	3.70 ± 1.13	4.14 ± 0.93	−0.44 [−0.94, 0.05]	0.43 [− 0.05, 0.90]	0.077

*FEIRT-CIC-SF* Facial Emotion Intensity Rating Test–Congruent and Incongruent, shortened five-emotion version, *ALS* amyotrophic lateral sclerosis, *pure motor ALS* ALS without cognitive and behavioural impairment according to the revised Strong criteria, as assessed with the ECAS, with normal performance in all ECAS cognitive domains, and without clinically relevant depressive symptoms or other psychiatric disorders. *HC* healthy controls, *ECAS* Edinburgh Cognitive and Behavioural ALS Screen, *NET* negative emotion total. Values are mean ± SD. Mean differences are reported as ALS − HC. d = Cohen’s d (standardised mean difference; positive values indicate higher scores in HCs). All confidence intervals are two-sided 95% CIs, and all p-values are from two-sided Welch’s t tests. Individual emotion comparisons were exploratory; p-values are uncorrected (Holm-adjusted across the four negative emotions: anger 0.026, fear 0.039, disgust and sadness 0.615). NET-Con and NET-Incon are the congruent and incongruent components of the NET-Contrast score (higher values indicate higher intensity ratings). Individual emotion contrasts were computed per facial expression (congruent rating minus the mean of the incongruent ratings on the three other negative emotions for the same facial stimuli). The NET-Contrast equals the mean of the four individual emotion contrasts

Considered separately, congruent and incongruent ratings did not differ significantly between patients and HCs (t(67.9) = −1.51, p = 0.137, and t(61.0) = 1.54, p = 0.128, respectively; Table 2).

### Post-hoc analyses: ability to distinguish a negative emotion from other negative emotions

Exploratory post-hoc analyses of the individual negative-emotion contrast scores yielded group differences for anger and fear (Holm-adjusted p = 0.026 and p = 0.039), with no significant differences for sadness or disgust (Table 2). The group × emotion interaction was not significant (F(3, 204) = 1.65, p = 0.178, partial η^2^ = 0.024), providing no evidence that the ALS–HC difference varied across negative emotions. As HCs hardly distinguished disgust from the other negative emotions (Disgust-Contrast: M = 0.25, 95% CI [−0.32, 0.82]), the analysis was repeated post-hoc without disgust, with similar results (group × emotion interaction p = 0.179; group difference across the three remaining emotions, Welch’s t test, p = 0.005).

### Ability to distinguish happiness from the four negative emotions

Happiness-Contrast scores did not differ significantly between patients and HCs, although the effect size was small to medium (t(65.6) = −1.80, p = 0.077, Cohen’s d = 0.43, 95% CI [−0.05, 0.90]; Table 2).

A 2 × 2 mixed ANOVA comparing the NET-Contrast and Happiness-Contrast scores between groups showed no significant group × valence interaction (F(1, 68) = 0.56, p = 0.459, partial η^2^ = 0.008); thus, there was no evidence that the ALS–HC difference was larger for discrimination among negative emotions than for discrimination of the positively valenced emotion, happiness, from negative emotions.

### Ability to discriminate non-emotional facial features

FSMT scores did not differ significantly between groups (Wilcoxon rank-sum test, W = 585, p = 0.725; Table 1), with performance close to ceiling in both (maximum score: ALS 54%, HC 57%).

## Discussion

Our study demonstrates impaired discrimination of negative facial emotions in patients with pure motor ALS compared with HCs. To our knowledge, based on two recent systematic reviews [14, 17] and further studies not included in these reviews [21, 43], this is the first study to examine facial emotion discrimination in patients with ALS who were classified as pure motor according to the revised Strong criteria, with the additional requirements of normal performance in the cognitive functions that do not define ALSci under these criteria (memory and visuospatial functions) and the absence of clinically relevant depressive symptoms as defined by an a priori cut-off on an ALS-specific depression instrument. The group difference was not significantly larger for negative emotions than for happiness; our findings, therefore, do not establish a deficit confined to negative emotions.

With respect to overall ER performance, these results are consistent with the two previous cohorts of ALS patients without cognitive or behavioural impairment, which were examined with different ER tasks: the EK-60F [27] in the study of Palumbo et al. [20] and the CATS in the largely overlapping samples of Castelnovo et al. [21, 44]. Both reported poorer overall performance in patients than in HCs, with effect sizes similar to ours (Palumbo et al.[20]: Cohen’s d = 0.82, 95% CI [0.39, 1.24]; Castelnovo et al. [21]: d = 0.56, 95% CI [0.08, 1.04], for the CATS total score, which includes a non-emotional identity discrimination subtest; d values for the EK-60F and CATS scores calculated from the published group means and standard deviations; present study: d = 0.63, 95% CI [0.14, 1.11]). The individual emotions reaching significance, however, differed between cohorts: sadness and fear in the study of Palumbo et al.[20], disgust only in the samples of Castelnovo et al. [21, 44], and anger and fear in our exploratory analyses. None of the studies showed that the impairment differed between emotions: this was not tested in the previous studies, and the corresponding interaction was not significant in ours. In our study and in that of Castelnovo et al. [21], a non-emotional face task (FSMT and CATS identity discrimination, respectively) showed no significant group difference, although both tasks were close to the ceiling.

Several methodological differences may contribute to this variation. First, the cohorts were defined differently: both previous studies classified patients according to the revised Strong criteria, in which cognitive impairment is defined by executive (including social-cognitive) and language dysfunction and isolated memory impairment does not qualify as ALSci [3]; we additionally required normal memory and visuospatial performance, the ALS-non-specific domains of the ECAS [10]. Second, depression was handled differently: Palumbo et al. excluded patients with major depression [20], and Castelnovo et al. reported that no patient showed clinical depression on rating scales or clinical interview, without stating a cut-off [21, 44], whereas we excluded clinically relevant depressive symptoms on the basis of a predefined cut-off on an ALS-specific instrument (ADI-12 ≥ 29) [24, 25]. Third, our primary analysis focused on the four negative emotions, whereas the previous studies evaluated all six. Fourth, the tasks differ substantially: the EK-60F and the CATS present non-morphed, full-intensity Ekman faces with categorical response formats, which carry a risk of ceiling effects in healthy individuals, whereas the FEIRT-CIC uses morphed expressions at threshold intensity, rated on a 7-point scale, to avoid them [22]. Finally, the absence of a disgust effect in our study, in contrast to the studies of Castelnovo et al. [21, 44], may partly be related to the relatively low discriminability of disgust in our task even among HCs.

Regarding potential clinical utility, the FEIRT-CIC may offer advantages over tasks using full-intensity Ekman faces with categorical responses, such as the EK-60F used by Palumbo et al. [20] and the CATS used by Castelnovo et al. [21], which should be evaluated in future validation studies. Its graded (7-point) intensity ratings [26] and morphed stimuli free of ceiling effects [22] may afford greater sensitivity to graded impairments, although effect sizes were similar across the three cohorts. In addition, rating one named emotion at a time should reduce the working-memory demands of selecting among competing verbal labels [45], potentially providing a purer index of perceptual emotion discrimination. The FEIRT-CIC-SF used here takes approx. 20 min [46], compared with approx. 30 min for the original FEIRT-CIC [26]; a version restricted to the four negative emotions (32 trials) would take an estimated 10–15 min.

Our findings are compatible with the conceptualisation of ALS and FTD as parts of one spectrum (ALS-FTSD). Within this spectrum, some patients present predominantly with motor features, whereas ALS-FTD represents a phenotype with prominent cognitive and/or behavioural involvement. Previous studies have shown that ER deficits are particularly pronounced in ALS-FTD [17] and bvFTD [18, 19]. In the present study, we found impaired facial emotion discrimination even in patients with pure motor ALS. As our study did not include ALSci, ALSbi, or ALS-FTD comparison groups, however, it cannot determine whether impaired emotion discrimination relates to additional cognitive or behavioural impairment.

Patients with ALS-FTD present with progressive behavioural and/or cognitive deterioration meeting diagnostic criteria adapted from those for bvFTD [3, 38], or with language impairment fulfilling the criteria for the semantic or non-fluent variant of primary progressive aphasia [3, 47], with the behavioural variant being the typical FTD phenotype in ALS [48]. Evidence indicates that: (i) ER deficits are most pronounced in patients with ALS-FTD compared to other ALS phenotypes [17], (ii) ER impairment is severe in patients with bvFTD [19], and (iii) ALS-FTD and bvFTD show ER deficits of comparable severity [18]. Notably, ER deficits are a prominent feature of bvFTD [19], and subtle changes have been reported in presymptomatic C9orf72 mutation carriers [49]. Consistent with this, in a previous study using the original FEIRT-CIC, we demonstrated that individuals in the mild stages of bvFTD are profoundly impaired in distinguishing between negative facial emotions [26]. 

The group difference was of medium size (Cohen’s d = 0.63), and the distributions overlapped considerably (Fig. 1b): 26 of the 35 patients (74%) scored below the HC median, but only 5 (14%) scored below the lowest HC score. Such overlap, together with small samples and the known heterogeneity of extra-motor involvement across ALS-FTSD [1, 3], may also explain why some previous studies found no ER deficits in ALS patients without dementia [18] or without behavioural symptoms [50].

Against this background of heterogeneous social-cognitive involvement, our findings also carry implications for the design of cognitive screening tools in ALS. Current ALS-specific cognitive screening batteries, such as the ECAS, do not include a dedicated ER subtest. Instead, social cognition is represented by an adapted version of Baron-Cohen et al.’s Judgement of Preference based on eye gaze [51], embedded within the "Executive Functions" domain of the ECAS [10]. However, ToM is a higher-order cognitive ability closely intertwined with executive components [13], whereas ER deficits can occur independently of executive dysfunction [16]. An ER-based measure may, therefore, index social cognition more specifically, with less confounding by executive demands, than a ToM paradigm. In our sample, patients did not score lower than HCs on the ECAS ToM subtest (Supplementary Material C), but showed impaired emotion discrimination. More generally, the finding that patients classified as pure motor according to the revised Strong criteria, operationalised using the ECAS and with normal performance in all its cognitive domains, nonetheless showed impaired emotion discrimination suggests that classification based on ECAS screening, which contains no ER task, may underestimate social-cognitive involvement in ALS. Under the revised Strong criteria, moreover, an isolated social-cognitive deficit does not qualify as cognitive impairment [3]. Our finding is in line with reports of ER impairments even in cognitively preserved ALS patients [16, 20], and with the observation that errors in dynamic, multimodal ER in ALS were not associated with ECAS-defined cognitive impairment [52]. For these reasons, ER tasks merit evaluation as candidates for representing social cognition in future ALS screening instruments; their diagnostic value, however, remains to be established.

An important qualification of the phenotype of our cohort is that ALS patients underwent only a brief multi-domain, ALS-specific assessment using the ECAS [10], rather than a comprehensive cognitive assessment. Nevertheless, the ECAS has been shown to perform well in comparison with a comprehensive neuropsychological assessment in determining ALSci [53]. Our classification corresponds to Level I of the revised Strong criteria [3], and social cognition is represented in the ECAS by a single subtest; taken alone, this subtest identified 38% of patients with ALS-specific cognitive impairment (specificity 100%) [53], and its sensitivity to social-cognitive impairment itself has not been established. Furthermore, the comparisons of individual emotions were exploratory, as the study was not powered to detect smaller differences in impairment between them, and require replication in larger, independent cohorts. The cross-sectional design of the present study precludes conclusions regarding the longitudinal trajectory of ER deficits in pure motor ALS. Whether ER impairment emerges prior to, or progresses independently of, deficits in other cognitive domains – and whether it may serve as an early marker of subsequent cognitive decline within ALS-FTSD – remains to be established in future longitudinal studies. Given the few trials, near-ceiling performance, and modest reliability of the FSMT, subtle differences in basic facial processing cannot be excluded. All HCs were recruited in Basel, so that site and group were partially confounded. The sensitivity analyses show that the result does not depend on pooling patient sites, but a site effect cannot be excluded; test administration was standardised across sites, and the HC mean matched that of an earlier sample from the same participant pool[26]. In addition, subthreshold depressive symptoms were not excluded in patients; in an earlier study with the original FEIRT-CIC, however, patients with major depressive disorder had higher, not lower, NET-Contrast scores than healthy participants [26]. Finally, examiners were not blind to the diagnostic group.

In conclusion, our findings emphasise the relevance of evaluating ER as a key component of social cognition in ALS. They suggest that facial emotion discrimination may capture subtle social-cognitive differences in ALS that are not reflected by established ALS-specific cognitive screening measures. Deficits in emotion processing may have important consequences for patient–carer interactions and family stress [7], and greater clinical awareness of socio-emotional changes in ALS may help clinicians and carers recognise and address related challenges in daily life. Future studies in independent samples need to determine the sensitivity, specificity, clinically meaningful cut-offs, test–retest reliability, incremental diagnostic value, and longitudinal predictive validity of the FEIRT-CIC and its shortened versions.

## Supplementary Information

Below is the link to the electronic supplementary material.

Supplementary file1 (DOCX 1762 KB)

## Data Availability

The datasets analysed during the current study are not publicly available owing to privacy regulations governing sensitive patient information (Swiss Federal Act on Data Protection, nDSG). Anonymised data can be shared with qualified researchers on reasonable request to the corresponding author, subject to a data transfer agreement.
