# Supplementary material for: Impaired discrimination of negative facial emotions in pure motor ALS
Source: J Neurol. 2026 Nov 10;273(11):709. doi: 10.1007/s00415-026-14241-x (PMC13751581; doi:10.1007/s00415-026-14241-x)
Supplement: Supplementary file 1 [file 415_2026_14241_MOESM1_ESM.docx]

**Supplementary material**

**Impaired discrimination of negative facial emotions in pure motor ALS**

Antonia Meyer^1,2^, Anna Semenkova^1,3^, Steven Wezel^1,2,3^, Markus Weber^4^, Kathi Schweikert^2^, Regina Schläger^2^, Janina Wendebourg^2^, Olivier Piguet^5^, Manfred Berres^6^, Nathalie Braun^4^, Dorothée Lulé^7^ and Marc Sollberger^1,2^

1	Memory Clinic, Department of Dementia Diseases, University Hospital of

Geriatric Medicine FELIX PLATTER and University of Basel, Basel, Switzerland

2	Department of Neurology and Department of Clinical Research, University Hospital Basel, Basel, Switzerland

3	Faculty of Psychology, University of Basel, Basel, Switzerland

4	HOCH Health Ostschweiz, Kantonsspital St. Gallen Muskelzentrum / ALS Clinic, St. Gallen, Switzerland

5	The University of Sydney, School of Psychology and Brain and Mind Centre, NSW, Australia

6	Department of Mathematics and Technology, University of Applied Sciences Koblenz, Germany

7	Department of Neurology, Ulm University Medical Center, Ulm, Germany

**Supplementary Material A. Power analysis plan	3**

**A.1. Determination of sample size	3**

**A.2. Classic Faces Test for bvFTD	3**

**A.3. Novel Faces Test for bvFTD	4**

**A.4. Classic Faces Test for ALS	4**

**A.5. Anticipated Effect Size of ALS on Novel Faces Test	4**

**A.6. Required Number of Observations	5**

**A.7. Primary analysis	5**

**Supplementary Material B. Psychometric Analyses of the Facial Sex Matching Task in 240 healthy adults	6**

**Supplementary Material C. ECAS Theory of Mind subtest performance in pure motor ALS patients and healthy controls	8**

**Supplementary Material D. NET-Contrast scores by recruitment site	9**

**Supplementary Material E. Slides of the Facial Sex Matching Task	10**

**Supplementary Table S1. Additional clinical characteristics of the ALS patients	14**

**Supplementary Figure S1. Participant flow diagram	15**

**Supplementary Material A. Power analysis plan**

**A.1. Determination of sample size**

This study aims to show that the adapted test is able to detect an effect that has already been shown in the literature using the classic test. Therefore, we use the literature to derive an estimate of the expected effect size. The logic of this derivation is as follows.

1. There are studies comparing ALS patients to healthy controls using the previous facial emotion perception test.
2. There are studies comparing bvFTD patients to healthy controls, both using the previous facial emotion perception test and the novel adaptation that will be applied in this study.
3. From these, we can define the ratio of the sensitivity of the adapted test and the original test for showing impaired emotional perception of bvFTD patients.
4. Combining this ratio with the observed ALS effect on the classic test, we can derive an informed guess about the effect size of ALS on the novel adapted test to be observed in the current study.

**A.2. Classic Faces Test for bvFTD**

Here, we derive the observed standardised effect size (Cohen’s d) of the classic FEEST

emotional faces test for bvFTD patients from two studies[1, 2].

In the first study[1], the mean faces scores for bvFTD patients are compared to those of the healthy control group. However, since the primary analysis of the adapted version is based on negative faces, it makes more sense to consider only the scores of negative faces. Therefore, we take the mean scores on the 4 negative emotions for both bvFTD patients and healthy controls and subtract those to get 4 difference scores. The average of these difference scores is 1.04. We pool the standard deviations of the 4 scores for both bvFTD patients and healthy controls, yielding an average SD of 1.84. These numbers combined offer an estimate of the effect size δ of bvFTD vs HC (healthy controls) on the perception of negative emotions in the classic faces test of 1.04/1.84 = 0.56. The second study[2] offers rather similar numbers.

Following the same procedure, we derive the average effect in this study to be 7.56. Given an average SD of 17.25, this yields an estimated effect size δ of 7.56/17.25 =

0.44. Averaging the effect sizes of these two studies, we estimate the effect size δ of bvFTD

vs HC on the negative emotions in the classic faces test to be 0.5.

**A.3. Novel Faces Test for bvFTD**

Now, we assess the observed standardised effect size (Cohen’s d) of bvFTD vs HC on the perception of negative emotions in the adapted faces test, i.e., the Facial Emotion Intensity Rating Test – Congruent and Incongruent. This assessment is based on the paper by Chiu et al. (2018)[3].

In this study, the mean score for bvFTD patients is 0.26, and 2.5 for HCs. The pooled standard deviation is 1.08. So, the effect size estimated based on this article is 2.24/1.08 = 2.07.

**A.4. Classic Faces Test for ALS**

Here, we assess the standardised effect size (Cohen's d) of ALS vs HC on the negative emotions in the classic faces test. The statistics in this paragraph are based on two studies: Oh et al.[4] and Aho-Özhan et al.[5].

In Oh et al.[4], the mean score of ALS patients across the four negative emotions is 53.1, and for HCs 66.3. The pooled standard deviation is 14.28. These numbers yield an effect size of 13.2/14.28 = 0.92.

In Aho-Özhan et al.[5], the mean score of ALS patients across the four negative emotions is 5.68, and for HCs 6.88. The pooled standard deviation is 2.23. These numbers yield an effect size of 1.20/2.23 = 0.54. Averaging the effect sizes of these two studies, we estimate the effect size of ALS vs HC on the negative emotions in the classic faces test to be 0.73.

**A.5. Anticipated Effect Size of ALS on Novel Faces Test**

Combining the values calculated above: δALS,novel = 2.07/0.5 × 0.73 = 3.02. However, this estimate is likely a substantial overestimate of the true effect for three reasons. First, both reference ALS studies included clinically heterogeneous samples that were not restricted to pure motor ALS patients — patients with concurrent cognitive and behavioural impairments were not systematically excluded, which inflates the effect size relative to what would be expected in a strictly defined pure motor cohort[4, 5]. Second, the formula assumes that the relative sensitivity advantage of the novel over the classic task is the same for ALS as for bvFTD. Given that bvFTD shows profoundly more severe emotion recognition deficits than pure motor ALS[6, 7], this assumption likely does not hold, and the amplification factor (2.07/0.5 = 4.14) derived from bvFTD data is unlikely to transfer directly to the subtler deficits expected in the present sample of ALS. Third, the reference ALS studies differed in their emotion recognition instruments: Oh et al.[4] used a Korean-validated tool (ChaeLee-E), limiting cross-cultural comparability with the FEIRT-CIC, while Aho-Özhan et al.[5] used the FEEST, which shares the same stimulus material as the FEIRT-CIC but employs a fundamentally different response format. For these reasons, the formula-derived estimate of δ = 3.02 was regarded as an upper bound rather than a realistic expectation. Rather than relying on this inflated value, we anchored our pre-specified effect size conservatively on the empirically observed effect of ALS in the classic test (δ = 0.73; see A.4), which reflects the actual magnitude of emotion recognition impairment reported in ALS cohorts rather than an extrapolation from bvFTD. Accordingly, a conservative anticipated effect size of δ = 0.75 was pre-specified. Based on a one-tailed test at α = 0.05, this yields a target sample of n = 35 per group, providing >90% power; this estimate is further strengthened by the age-, sex- and education-matched design.

**A.6. Required Number of Observations**

Given the pre-specified effect size of δ = 0.75 (conservatively anchored on the classic-test ALS effect; see A.5), a one-tailed two-sample t-test at α = 0.05 with n = 35 per group (N = 70) yields a statistical power of 0.93.

**A.7. Primary analysis**

The required sample size reported above is calculated assuming that a two-sample t-test will be performed. The two groups compared in this test are the ALS patients’ group and the healthy control group. The two-sample t-test will be performed on the contrast measure of the FEIRT-CIC-SF (shortened five-emotion version of the FEIRT-CIC), one-sided, at an alpha level of 0.05. Following peer review, all analyses are reported two-sided; for a two-sided test, the corresponding power is 0.87.

**Supplementary Material B.**

**Psychometric Analyses of the Facial Sex Matching Task in 240 healthy adults**

The psychometric evaluation of the Facial Sex Matching Task (FSMT) (originally termed Gender Discrimination Task) was conducted in a sample of 240 cognitively and mentally healthy adults (121 female, 119 male), evenly distributed across five age groups (35–44, 45–54, 55–64, 65–74, and 75 years or older; age range 34–92 years)[8].

All participants completed a baseline assessment, and a 12-month follow-up assessment was planned; however, only 72 participants (30%) returned for the follow-up. The FSMT was administered as a computer-based task using morphed Ekman faces with neutral expressions derived from the Facial Expressions of Emotion – Stimuli and Tests[9]. In each trial, two neutral faces were presented side by side, depicting either the same or different sexes, and participants indicated whether the faces were of the same sex. Following one practice item, six test trials were administered in a fixed order. Stimuli remained on screen until a response was given, and the task was untimed. Performance was quantified as the number of correct responses across the six trials (range 0–6). Please find the test in the Supplementary Material E.

Performance across the sample was highly skewed toward the maximum score and demonstrated clear ceiling effects (see Figure B1). The median total score was 6 (mean: 5.77, min value: 2, max value: 6, standard deviation: 0.62), representing the maximum number of correct responses. A total of 205 participants (85.4%) solved all items correctly. Nineteen participants (7.9%) made one error, 13 participants (5.4%) made two errors, two participants (0.8%) made three errors, and one participant (0.4%) committed four errors. Thus, deviations from maximum performance were uncommon, and error patterns were sparsely distributed.

Test–retest correlation was modest with r = .68. Internal consistency was modest, with Cronbach’s alpha ranging between .55 and .62. The intraclass correlation coefficient (ICC = .46) indicated fair reliability. Validity evidence was limited: content validity was judged on logical grounds only, construct validity could not be examined for lack of a comparable test, and criterion validity was considered only partially met.

In summary, the psychometric profile of the FSMT is limited by its modest reliability and unexamined construct validity. It may nevertheless serve as a brief screen for marked deficits in the perception of non-emotional facial features in patients scheduled for an FEIRT-CIC assessment, which is another instrument that uses morphed stimuli from the FEEST[9].

**Figure B1**. Distribution of the Facial Sex Matching Task sum score (0–6) in 240 healthy adults

Sum score of Facial Sex Matching Task

Frequency

**Supplementary Material C.**

**ECAS Theory of Mind subtest performance in pure motor ALS patients** **and healthy controls**

This supplementary material reports group comparisons on the ECAS Theory of Mind subtest, an adapted version of Baron-Cohen et al.'s Judgement of Preference based on eye gaze[10], embedded within the Executive Functions domain of the ECAS[11]. Scores were close to ceiling in both groups, which limits the sensitivity of this subtest in the present sample. Because the normality assumption was violated in both groups — a pronounced ceiling effect in the ALS group and a bimodal distribution in controls (a ceiling cluster plus a small low-scoring subgroup) — we carried out a Wilcoxon rank-sum (Mann–Whitney U) test. The maximum score of 12 was reached by 94% of ALS patients and 77% of healthy controls, so that both group medians were 12 (IQR 12–12); the means, however, exposed the underlying divergence (ALS: M = 11.94, SD = 0.24, range 11–12; HC: M = 10.26, SD = 3.48, range 2–12). This analysis revealed a statistically significant, yet clinically counterintuitive, difference, with the ALS group scoring higher than controls (tie-corrected Mann–Whitney U = 500.5, z = 2.15, p = .031, rank-biserial r = 0.18 – a small effect; r computed per Kerby 2014[12], thresholds per Cohen 1988[13]; n = 35 per group), driven by a small low-scoring subgroup of controls rather than by superior Theory of Mind performance in the patients.

**Supplementary Material D.**

**NET-Contrast scores by recruitment site**

Within the ALS group, NET-Contrast scores differed between the three recruitment sites (Basel: M = 3.20, SD = 0.46, n = 4; St. Gallen: M = 1.33, SD = 0.73, n = 9; Ulm: M = 1.75, SD = 1.00, n = 22; Welch’s one-way ANOVA, F(2, 11.54) = 16.84, p < .001; Kruskal–Wallis χ²(2) = 8.25, p = .016). Patients from St. Gallen and Ulm did not differ (Welch’s t(20.43) = −1.31, p = .204), whereas the four patients from Basel scored above the HC mean (M = 2.45). The group difference was preserved versus all HCs (n = 35) when restricting the patient sample to Ulm (n = 22; mean difference = −0.70, 95% CI [−1.25, −0.15], Welch’s t(45.50) = −2.54, p = .014, Cohen’s d = 0.69) or excluding patients from Basel (n = 31; mean difference = −0.82, 95% CI [−1.30, −0.34], Welch’s t(63.91) = −3.40, p = .001, Cohen’s d = 0.83). As all HCs were recruited in Basel, site and group cannot be formally separated; test administration was standardised across sites.

**Supplementary Material E.**

**Slides of the Facial Sex Matching Task (Test zum Geschlechtsvergleich von Gesichtern)**

The faces are taken from the Facial Expressions of Emotion – Stimuli and Tests (FEEST)[9]. The slides are shown in the order of presentation (German original). English translation: “Instruction: In this task, you will be shown two faces at a time. Please decide whether the two faces are of the SAME SEX. You can make your decision by mouse click or tell the examiner.” – “A practice trial follows.” – “Is this the same sex? Yes / No.” – “The task begins now.” – “The task has ended.”

|  |  |
| --- | --- |
|  |  |
|  |  |
|  |  |
|  |  |
|  |  |

**References**

1.	Diehl-Schmid J, Pohl C, Ruprecht C et al (2007) The Ekman 60 Faces Test as a diagnostic instrument in frontotemporal dementia. Arch Clin Neuropsychol 22:459–464. https://doi.org/10.1016/j.acn.2007.01.024

2.	Fernandez-Duque D, Black SE (2005) Impaired recognition of negative facial emotions in patients with frontotemporal dementia. Neuropsychologia 43:1673–1687. https://doi.org/10.1016/j.neuropsychologia.2005.01.005

3.	Chiu I, Piguet O, Diehl-Schmid J et al (2018) Facial emotion recognition performance differentiates between behavioral variant frontotemporal dementia and major depressive disorder. J Clin Psychiatry 79:16m11342. https://doi.org/10.4088/JCP.16m11342

4.	Oh S, Oh K-W, Kim H-J et al (2016) Impaired perception of emotional expression in amyotrophic lateral sclerosis. J Clin Neurol 12:295. https://doi.org/10.3988/jcn.2016.12.3.295

5.	Aho-Özhan HEA, Keller J, Heimrath J et al (2016) Perception of emotional facial expressions in amyotrophic lateral sclerosis (ALS) at behavioural and brain metabolic level. PLoS One 11:e0164655. https://doi.org/10.1371/journal.pone.0164655

6.	Carelli L, Solca F, Tagini S et al (2021) Emotional processing and experience in amyotrophic lateral sclerosis: a systematic and critical review. Brain Sci 11:1356. https://doi.org/10.3390/brainsci11101356

7.	Kumfor F, Piguet O (2012) Disturbance of emotion processing in frontotemporal dementia: a synthesis of cognitive and neuroimaging findings. Neuropsychol Rev 22:280–297. https://doi.org/10.1007/s11065-012-9201-6

8.	Frei C (2021) Psychometrische Analyse des Geschlechtsdiskriminationstests (Gender discrimination task). Unpublished Master’s thesis, Universität Basel, Fakultät für Psychologie

9.	Young A, Perrett DI, Calder A et al (2002) Facial expressions of emotion: Stimuli and Tests (FEEST). Thames Valley Test Company, Bury St Edmunds

10.	Baron-Cohen S, Campbell R, Karmiloff-Smith A et al (1995) Are children with autism blind to the mentalistic significance of the eyes? Br J Dev Psychol 13:379–398. https://doi.org/10.1111/j.2044-835X.1995.tb00687.x

11.	Abrahams S, Newton J, Niven E et al (2014) Screening for cognition and behaviour changes in ALS. Amyotroph Lateral Scler Frontotemporal Degener 15:9–14. https://doi.org/10.3109/21678421.2013.805784

12.	Kerby DS (2014) The simple difference formula: an approach to teaching nonparametric correlation. Compr Psychol 3:11.IT.3.1. https://doi.org/10.2466/11.IT.3.1

13.	Cohen J (1988) Statistical power analysis for the behavioral sciences, 2nd edn. Lawrence Erlbaum Associates, Hillsdale, NJ

**Supplementary Table S1.**

**Additional clinical characteristics of the ALS patients (*n* = 35)**

|  | **Pure motor ALS** |
| --- | --- |
|  | **n = 35** |
| **Time from symptom onset to assessment (years)^a^** | 1.96 ± 1.53 |
| **Site of initial motor involvement, n (%)** |  |
| Bulbar involvement at onset | 10 (29) |
| Spinal-only onset | 25 (71) |
| Upper-limb onset | 14 (40) |
| Lower-limb onset | 9 (26) |
| Mixed upper-/lower-limb onset | 2 (6) |
| **Non-invasive ventilation (NIV), n (%)** |  |
| Yes | 7 (20) |
| No | 28 (80) |
| **ALSFRS-R respiratory subscore (0–12), mean ± SD^a^** | 10.23 ± 2.67 |
| **ALS-specific medication, n/N (%)**^b^ |  |
| Riluzole | 27/31 (87) |
| Edaravone | 7/31 (23) |
| **Genetic status, n (%)** |  |
| C9orf72 repeat expansion documented | 2 (6) |
| Unavailable / not assessed | 33 (94) |
| **Family history of ALS, n (%)** |  |
| None | 31 (89) |
| Positive | 3 (9) |
| Unknown | 1 (3) |
| ^a^n = 33. ^b^Medication data were extracted from free-text medication records; medication entries were available for 31/35 participants. | |

ALS = Amyotrophic Lateral Sclerosis, pure motor ALS = ALS without cognitive and behavioural impairment according to the revised Strong criteria, as assessed with the ECAS, with normal performance in all ECAS cognitive domains, and without clinically relevant depressive symptoms or other psychiatric disorders. Motor phenotype was not systematically classified; therefore, only the site of initial motor involvement is reported. Respiratory status is reported as use of non-invasive ventilation and the ALSFRS-R respiratory subscore. Percentages may not sum to 100 because of rounding.

**Supplementary Figure S1.**

**Participant flow diagram**
